# Stem cell-based therapies for treatment of abdominal aortic aneurysm: development, application, and future potential

**DOI:** 10.1038/s44385-025-00044-8

**Published:** 2025-11-26

**Authors:** Seleem Badawy, Shivesh Anand, Ande X. Marini, Joscha Mulorz, Philip S. Tsao, Ngan F. Huang

**Affiliations:** 1 Department of Cardiothoracic Surgery, Stanford University, Stanford, CA USA; 2 Center for Tissue Regeneration, Repair and Restoration, Veterans Affairs Palo Alto, Health Care System, Palo Alto, CA USA; 3 Division of Cardiovascular Medicine, Stanford University, Stanford, CA USA; 4 Department of Vascular and Endovascular Surgery, University Hospital Düsseldorf, Heinrich-Heine University, Moorenstrasse 5, Düsseldorf, Germany; 5 Stanford Cardiovascular Institute, Stanford University, Stanford, CA USA; 6 Department of Chemical Engineering, Stanford University, Stanford, CA USA

**Keywords:** Cardiovascular biology, Mesenchymal stem cells, Pluripotent stem cells

## Abstract

Abdominal aortic aneurysms (AAA), with approximately 200,000 new diagnoses each year, represent a prevalent clinical concern. Current treatment includes monitoring and surgical procedures once the aneurysm reaches a certain size. However, the lack of effective, timely therapies leads to a high mortality rate due to rupture. With recent advancements and innovations in biomedical science, stem cell therapy has moved closer to widespread clinical use, with the field experiencing rapid growth since its inception in the late 20^th^ century. Given the pathophysiology of AAA, stem cell therapies have high potential impact in the treatment for both early and late-stage disease, targeting underlying mechanisms such as inflammation, vascular degeneration, and extracellular matrix degradation. There are many considerations and innovative potential approaches being explored in this type of treatment, such as strategically leveraging cell properties and their associated secretome and incorporating biomaterials-based strategies. This review article summarizes and critically assesses the efficacy of cell-based therapies in AAA preclinical models, current clinical trials in this area, and other emerging bioengineering approaches for the treatment of AAA.

## Introduction

Abdominal aortic aneurysm (AAA) is a vascular disease consisting of the abnormal dilation in the abdominal aorta diameter and simultaneous weakening of the aortic wall until rupture. AAAs have a prevalence of 150,000–200,000 new diagnoses each year^1^, with over 35 million active cases world-wide in 2019^2^. Often termed as the “silent killers”, the majority of AAAs are asymptomatic. As imaging has become broadly more common in healthcare check-ups, asymptomatic AAA have been increasingly identified earlier. Otherwise, AAAs tend to be discovered upon rupture, with a rupture mortality rate of approximately 80%^3^. AAAs are unique and vary in their morphology as well as progression among individuals. The heterogeneity of AAA and the ongoing challenges in diagnosis and understanding pathophysiology contribute to the challenge in both diagnostics and therapeutics of AAA.

Stem cell therapies, whereby stem cells are applied (systemically or locally) to repair tissues, have been an increasingly investigated possibility for AAA. With the term “stem cell” first coined in 1888, stem cell therapy has emerged as a field with major developments in the 20^th^ century^4^. The first role of stem cells in regenerative medicine was for the first bone marrow transplantation in 1956^5^. Important recent developments include the first isolation of mesenchymal stromal cells (MSCs) in 1991, especially relevant in the cardiovascular field, followed by the discovery of human pluripotent stem cells^4^. The therapeutic use of these stem cells enables and promises novel advancements in regenerative medicine, addressing the need for precision approaches to complex diseases. For AAA, stem cells can potentially address limitations of conventional treatments by targeting the underlying cellular and molecular mechanisms contributing to wall degradation, inflammation, and the other pathways occurring in the AAA cascade. Stem cell and other cell-based therapies aim to repair or regenerate damaged aortic tissue through various mechanisms, including processes improving regenerative, anti-inflammatory, and matrix-stabilizing effects. Cell-based therapies broadly present a unique advantage over current surgical interventions, which can only stabilize aneurysmal dilation at the late stage without reversing tissue degeneration. The long-term goal and potential of this therapy is to enable effective biological solutions to prevent further growth of AAA. With novel earlier-stage treatment options (currently non-existent in humans), this would dramatically improve both short and long-term outcomes for AAA care.

**Fig. 1:**
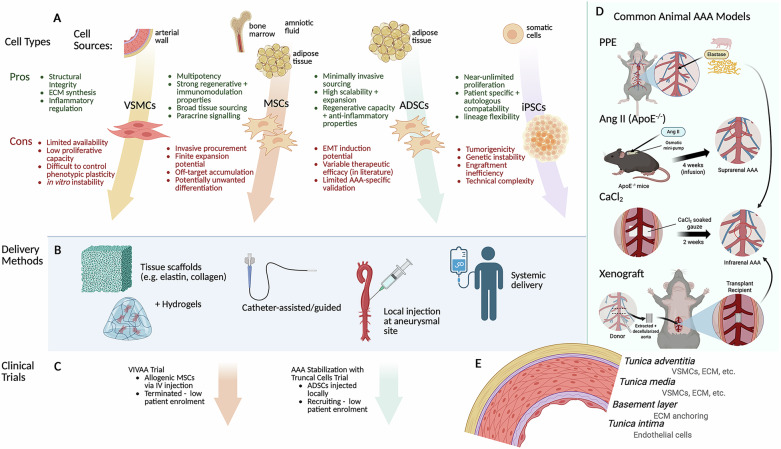
Schematic overview of key considerations in the design of cell therapies for treatment of AAA. **A** Therapeutic cell types, their respective sources, and other considerations. **B** Cell delivery modalities. **C** Status of relevant clinical trials. **D** Common animal models and their general techniques. **E** The aortic wall layers and relevant cells. VSMCs vascular smooth muscle cells, MSCs mesenchymal stromal cells, ADSCs adipose-derived stem cells, iPSCs induced pluripotent stem cells, ECM extracellular matrix, EMT epithelial-to-mesenchymal transition, AAA abdominal aortic aneurysm, IV intravenous, PPE porcine pancreatic elastase, Ang II angiotensin II, ApoE^−/−^ apolipoprotein E^−/−^ Created in BioRender. Badawy, S. (2025) https://BioRender.com/zf2ju2q.

There are many key considerations in the investigation and development of stem cell-based therapeutics (Fig. 1). The choice of stem cells (e.g., MSCs, adipose-derived stem cells (ADSCs), induced pluripotent stem cells, etc.) directly impacts the efficacy, sourcing, usability, and scalability, which are critical in developing effective technologies. The type of model for in vivo investigation, used for exploring the intricate effects of the different cells as well as possible delivery mechanisms, are also important to understand and establish the underlying mechanisms of the cells and their effects, as well as their limitations and implications for translation to humans. These, in addition to current pre-clinical and clinical studies, other approaches and developments, as well as future perspectives, will be discussed in this review to better understand the current state and promise of cell-based therapies for AAA treatment.

## AAA pathology

**Fig. 2:**
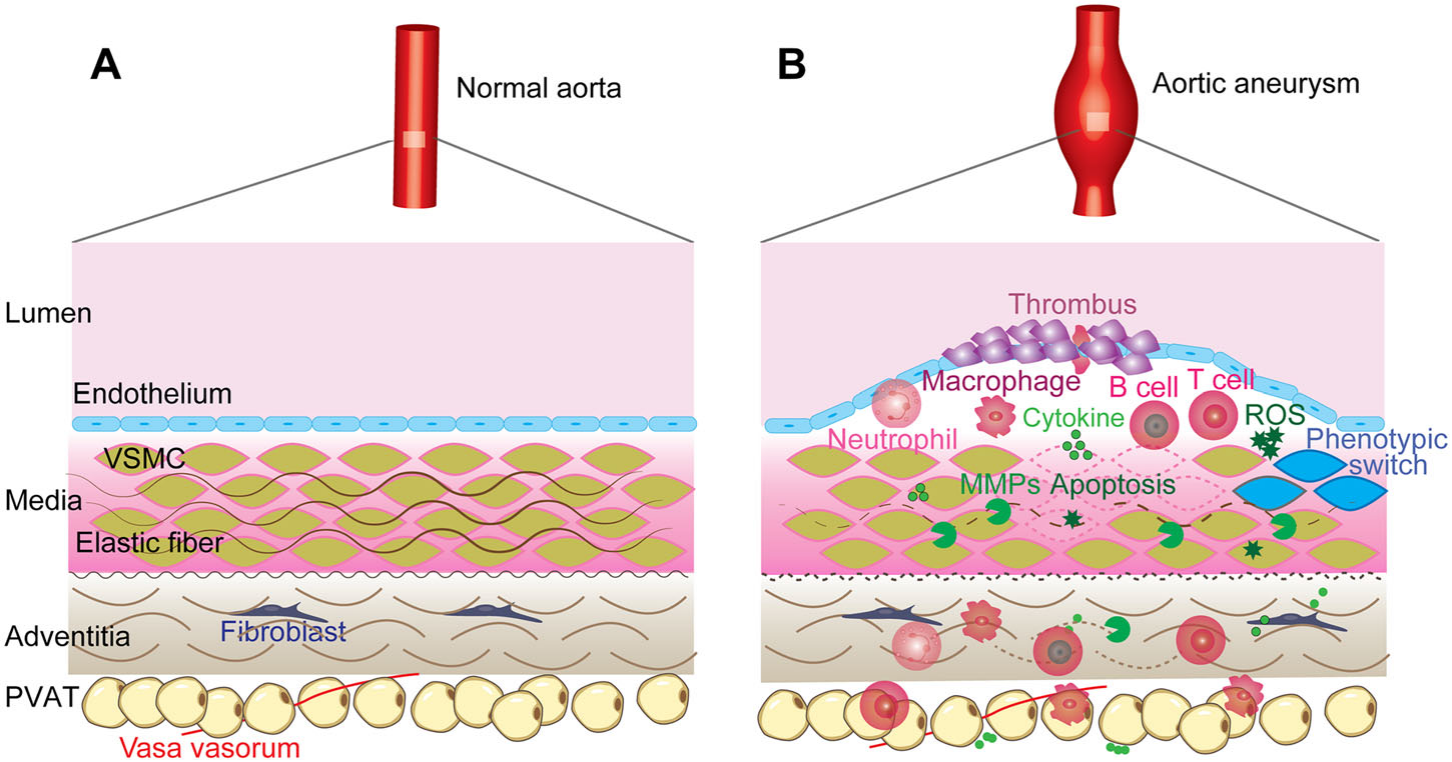
Pathological remodeling in AAA. **A** The healthy aorta cell environment, with surrounding perivascular adipose tissue (PVAT), compared to (**B**) the aortic aneurysm environment, characterized by thrombi, infiltration of inflammatory cells, ECM degradation, vascular smooth muscle cell (VSMC) phenotypic switching, apoptosis, excessive cytokine production, reactive oxygen species, and matrix metalloproteinases (MMPs). Reproduced with permission from Lu et al.^11^.

AAA pathophysiology is complex and consists of a cascade of multiple mechanisms with no known individual cause and reasonable contention surrounding the exact etiology (Fig. 2). The abdominal aorta contains VSMCs as a primary component of the medial layer that secrete extracellular matrix (ECM) proteins, such as elastin and collagen, as well as matrix metalloproteinases (MMPs)^6,7^. This allows for VSMCs to modulate the ECM for repair and degradation. There is evidence that VSMCs in AAA exhibit abnormal behaviour; a study by Airhart et al. shows that AAA VSMCs have greater elastolytic activity and MMP gene expression^6^. In AAA, inflammatory cells induce VSMC apoptosis, further reducing the structural integrity of the vessel and progressing the disease state^7^. High numbers of inflammatory macrophages, neutrophils, and helper T cells can be found in AAA lesions^8^. Studies have shown increased expression of proinflammatory transcription factors and cytokines produced by lymphocytes such as nuclear factor-κB (NF-κB), interleukin-6 (IL-6), and tumor necrosis factor α (TNF-α), some of which, when inhibited, have prevented aneurysm progression in animal models^8–10^. VSMCs also exhibit phenotypic switching from a quiescent, contractile phenotype (healthy) to a synthetic phenotype. In this synthetic state, the VSMCs release calcifying signals in the form of extracellular vesicles (EVs). They also move toward the site of injury and show increased proliferation^11,12^. This phenotypic switch occurs early in AAA development in both humans and mouse models^12,13^. Additionally, this synthetic/proliferative state has shown decreased SMC marker expression and increased phagocytic marker expression, in addition to upregulated MMP expression and downregulated contractile proteins, leading to vascular dysfunction and contributing to AAA pathogenesis^11,13,14^.

Moreover, many of the secreted factors by macrophage-like VSMCs form chronic inflammation contributing to the destruction of the aortic wall^14^. MMPs, which break down the ECM, have been shown to be present at high levels in AAA, specifically in their active forms and with MMP-2 and MMP-9 being particularly relevant in literature. These MMPs are likely contributed by and in response to local inflammatory cytokine production as well as VSMCs^6,8,15^. Tissue inhibitors of MMPs (TIMPs) are relevant as the negative modulator of MMP activity, both being key factors in AAA pathophysiology, especially in arterial wall integrity^16^. Oxidative stress and senescence are also relevant to the pathological conditions in AAA^11^. Due to vascular endothelial growth factor (VEGF) and other marker infiltration in the wall, angiogenesis has been observed at higher rates and contributes to the AAA environment, with neovascularization also being a critical contributor to AAA progression^17,18^. Ongoing research efforts focus on further understanding the exact mechanical and biological mechanisms and cascade that cause AAAs. Moreover, therapeutic and pharmacological approaches to AAA leverage or manipulate this complex disease environment to remediate AAA growth and progression.

## Aortic anatomy and possible targets for therapy

The aortic wall is composed of three tissue layers, the tunica intima, tunica media, and tunica adventitia, respectively from the lumen outwards. These are relevant to understanding the different cell source options and how they may interact with the aortic wall. The innermost intima layer is a thin monolayer of endothelial cells lining the vessel in direct contact with the blood, supported by a basement membrane^19^. The basement membrane is a highly specialized ECM structure that provides anchoring of the endothelial cells^20^. This intima layer is most susceptible to injury from atherosclerosis and aortic dissection. The middle “tunica media” layer is the thickest component of the aortic wall, particularly in arteries as opposed to veins^21^. It is comprised of mainly VSMCs, surrounded by a basal lamina and ECM with proteins including elastin and collagen, among connective tissue components like proteoglycans^22^. This layer is critical to structural integrity, withstanding the high pressure of the vessel, while retaining elasticity^21^; it is also responsible for maintaining intravascular pressure and tissue perfusion^23^. The VSMCs composing this layer are considered the most important cell type in AAA pathogenesis, with other cell types playing relatively minor roles^24^. The aortic VSMCs are able to contract, proliferate, and regulate extracellular matrix synthesis and degradation^23^. Given that VSMC dysfunction is prevalent to AAA, creating a stimulating/regenerative therapy for these cells could be a promising target. The outer adventitia layer is a thin layer composed mainly of fibroblasts and collagen fibers. It contains the vasa vasorum, microvessels that supply nutrients and oxygen to the outer layers of the larger vessel walls, and nerves^25^. This layer has the greatest tensile strength of the three layers due to its collagen-rich composition^25^.

## Initial VSMC therapies for AAA treatment

Since AAA is characterized by the loss of VSMCs, the therapeutic benefit of transplanted VSMCs has been tested in preclinical settings. Implementation of VSMCs for treatment of AAA (Table 1) utilized common experimental models to induce AAA including porcine pancreatic elastase (PPE), Angiotensin II (Ang II) and CaCl_2_ (Fig. 1D). Allaire et al. examined the effect of VSMC loss in the abdominal aorta on inflammation and formation of AAA. Using guinea pig xenografts seeded with rat VSMCs transplanted into male recipient rats, the authors showed macrophage infiltration into the autograft which is a hallmark of clinical AAA^26^. The implantation of xenografts seeded with VSMCs group were associated with a significant decrease in aneurysmal growth over time, with only a 35.3 ± 17.8% increase in mean aortic diameter 8 weeks after implantation, compared to xenografts with no cell seeding having a 198.2 ± 106.6% increase^26^. Animals treated with VSMC-seeded xenografts have also shown reduced elastin degradation, decreased monocyte-macrophage infiltration, and increased production of tissue inhibitor of matrix metalloproteinases (TIMPs), which contributed to reduced dilation^26^. This study found that MMPs may have been decreased by TIMPs, but was suggested to be decreased at the transcriptional level by VSMCs as well^26^. However, this study also pointed out that VSMCs alone could not completely prevent dilation^26^.

**Table 1:** VSMC therapies for AAA models

Disease model	Cell type	Delivery Method	Follow-up Time	Output Measurement	Reference
Guinea pig xenograft in Rats	Rat VSMCs	Seeded xenograft	Up to 56 days	VSMC prevented AAA formation, elastin degradation, and inflammatory response	^26^
Guinea pig xenograft in Rats	Rat SMCs	Injection into graft lumen	Up to 56 days	VMSC prevented further dilation of aneurysm and elastin degradation and inhibits MMP expression	^27^
Pancreatic porcine elastase infusion in Mice	Human iPSC smooth muscle progenitors or primary human VSMCs	Seeded porous collagen scaffold transplantation	Up to 28 days	VSMCs significantly decreased AAA growth more than iPSCs and had higher immune activity	^28^
Elastase induced AAA in Rats	VSMC-like progenitors derived from murine skeletal-muscle stem cells	Implantation into AAA lumen	Up to 42 days	VSMC-like progenitors had decreased AAA formation and inhibited MMP expression	^29^

*AAA* Abdominal Aortic Aneurysm, *MMP* Matrix Metalloproteinases, *Treg* Regulatory T Cells, *VSMC* Vascular Smooth Muscle Cells

In another study, the timing of VSMC delivery was tested in which the cells were injected into xenografts at two weeks post-surgery, during which the aneurysm had already started to form^27^. Eight weeks post-surgery, the VSMC group aneurysms remained relatively stable with limited growth in aneurysmal diameter and limited decrease in medial elastin content compared to the control group, without cells, and had a marked increase in aneurysmal diameter and decrease in elastin content^27^. Additionally, VSMC seeding showed a subsequent decrease in mononuclear infiltration as well as key MMP marker mRNA content in the diseased wall, while MMP-inhibitors increased^27^. Furthermore, VSMCs are sufficient to prevent further expansion in a pre-formed AAA model through prevention of further ECM degradation, notably in terms of elastin content, mainly via regulation of the MMP-dependent proteolytic balance in the aortic wall. Together, these animal studies provide evidence of VSMC therapy on significantly slowing AAA growth and restoring the healing capabilities of proteolytically injured ECM in vivo.

Bridging the path from VSMCs to stem cell-based therapies, the promise of iPSC-derived vascular smooth muscle progenitors, compared to primary human VSMCs, was shown by Mulorz et al. They investigated the localized delivery of both primary human-derived VSMCs and human iPSC-derived smooth muscle progenitors using porous collagen scaffolds to mice AAA with 5×10^5^ cells/scaffold^28^. Both the iPSC smooth muscle progenitors and VSMCs were validated as phenotypically similar, penetrated approximately 80% into the scaffold, and had no statistical difference in cell viability^28^. After 28 days from the AAA induction in the mice, the results indicate that the primary VSMC-seeded scaffold showed significantly decreased AAA growth compared to an acellular control and to the iPSC group (at 21 days)^28^. Moreover, 87% of the mice treated with the primary VSMC-seeded scaffolds were free from AAA compared to 33% in the iPSC-seeded group and no significant difference in AAA expansion in the acellular group^28^. This evidence supports primary VSMCs as a better candidate for AAA treatment than iPSCs and further demonstrates their promise in slowing AAA growth. VSMC-seeded scaffolds were also more immunologically active, with higher fold change increases across pro and anti-inflammatory cytokines compared to iPSCs, though both groups seemed to promote native SMC retention in the aortic *tunica media* layer^28^. Another study by Park et al. differentiated murine skeletal muscle-derived stem cells into VSMC-like progenitors and implanted these in the lumen of elastase-induced AAAs in rats (following enzymatic wall degradation)^29^. This cell therapy group again had a decreased rate of aneurysm formation compared to control groups, with significantly decreased MMP gene and protein expressions, especially MMP-9 and MMP-2^29^. Immunofluorescence imaging suggested that the VSMC-like progenitor cells inhibited the MMPs, thereby attenuating AAA formation^29^. These studies highlight the emerging area of smooth muscle progenitor or smooth muscle-like cells for treatment of AAA, giving rise to eventual stem cell-based therapies.

## Therapeutic stem cell types for AAA treatment

For the purpose of this review, we will focus on the most promising cell-based therapies and provide an update on the current state of this field. Stem cells identified for therapeutic use in AAA include MSCs, adipose derived stem cells, and induced pluripotent stem cell derivatives (Fig. 1A). Yamawaki-Ogata et al.^30^ provides a more comprehensive discussion of various cell-based therapies for AAA.

### Mesenchymal stromal cells (MSCs)

MSCs are multipotent cells known for their ability to differentiate into various cell types from the mesoderm, such as cartilage, bone, and adipose cells^31^. MSC populations can be extracted from multiple sources within the body including bone marrow, adipose tissue, and amniotic fluid^32^. Bone marrow-derived MSCs (BM-MSCs) are a popular source for cell therapy due to their availability, expandability in culture, and overall safety in clinical trials. Other types of MSCs include those derived from umbilical cords (UC-MSCs)^33^ and placental tissue (PL-MSCs)^34^. While no single phenotypic marker can characterize MSCs, these cells are generally thought to express phenotypic markers such as CD44, CD90, CD105, and CD73, as defined by the International Society for Cellular Therapy^35^.

In areas of tissue damage, MSCs are shown to release various cytokines and paracrine factors of potential therapeutic value^36^. In particular, MSC conditioned media was also shown to stimulate the proliferation of SMCs in vitro, likely through the expression of various cytokines and growth factors such as hepatocyte growth factor (HGF) or fibroblast growth factor-2 (FGF-2)^37^. Due to these immunosuppressive properties, MSC treatment may be beneficial to AAA as inflammation significantly contributes to aneurysm dilation and progression. MSCs also have a crucial role in AAA pathogenesis through dysregulation of their functional activities in the pathological environment, namely impaired immunomodulatory behavior, and increased levels of MMP-9^38^. MSCs have also been found to upregulate elastin and downregulate collagen expression in fibroblasts, participating in vascular injury remodeling^39^. These immunomodulatory pathways and regenerative properties of MSCs establish a strong molecular basis for the potential of MSCs to combat AAA progression at a cellular level.

Despite the potential of MSCs, some limitations of their use include: 1) MSC harvesting can be invasive and painful^40^; 2) although they have a high expansion ratio, MSCs cannot be expanded long-term^33^; and 3) their potential differentiation into osteogenic lineages is not desirable at the site of AAA. With respect to delivery, systemic delivery is less invasive but is prone to non-targeted effects to other organs (e.g. kidneys, spleen, etc.)^34,41^. Conversely, direct cell injection or implantation of a cell sheet is highly invasive despite its high targeting ability^39,42^. Endovascular delivery via catheter is less invasive with a high targeting ability^43,44^.

### Adipose-derived stem cells (ADSCs)

The potential of MSCs extends to ADSCs, proven to be an abundant, accessible and rich source of adult multipotent stem cells^45^. Although MSCs are commonly sourced from the bone marrow, particularly the iliac crest which produces a higher percentage of MSCs^46^, they can also be derived from adipose tissue using minimally invasive procedures like lipoaspiration^47^. ADSCs can be extracted from adipose tissue by collagenase digestion and then expanded in vitro^48^. Being isolated from more easily available subcutaneous fat, these cells can be rapidly acquired with high cellular activity^40^. Notably, ADSCs can be expanded in vitro for long periods of time while maintaining their differentiation capacity^40^, a stark difference from MSCs that favorably facilitates their practical use. Moreover, for both allogeneic and autologous applications, ADSCs have demonstrated optimal efficacy and efficiency^40^.

ADSCs show similar differentiation ability to MSCs, including immune regulatory properties. In the context of AAA, these cells demonstrate immunomodulatory and anti-inflammatory effects mediated by paracrine factors^49^. ADSCs were found to also increase select tissue-reparative markers^49^. They have also been used successfully in other illnesses including Parry-Romberg syndrome, Crohn’s disease, inflammatory and autoimmune disorders, and more^50^. Owing to the minimally invasive, less painful techniques to harvest these cells^40^, availability, and regenerative properties, ADSCs are an appealing cell source for therapeutic use, especially given their MSC-like properties. Other studies, however, have found that ADSCs can stimulate epithelial-mesenchymal transition and other challenges in cancer therapy uses and have shown previous negative results in cardiac disease treatment^50^, necessitating further clinical investigation of ADSCs for AAA.

### Induced pluripotent stem cells (iPSCs)

Induced pluripotent stem cells (iPSCs) hold significant promise as a novel cell source. iPSCs, derived from reprogrammed somatic cells, using only four transcription factors, have the capacity to differentiate into various cell types, including VSMCs or endothelial cells^51^. In AAA therapy, iPSC-derived cells can be used to repair or replace damaged aortic tissue, counteracting the inflammatory, apoptotic, and extracellular matrix-degrading processes characteristic of the disease. iPSCs have also been used as an effective in vitro cell model towards the development of aortic disease treatments^51^. Moreover, iPSCs provide a platform for patient-specific modeling of AAA pathophysiology and precision medicine options, generally serving as an unlimited cell source for lineage and patient-specific cells without many ethical concerns^52^. Though their large-scale production depends on the culture and differentiation, they are relatively more promising in this respect to other cell sources both for scalability and personalized medicine, with the capacity for almost unlimited expansion^53^.

The use of iPSC derivatives for treating abdominal aortic aneurysms offers several advantages, including their ability to generate patient-specific cells, reducing the risk of immune rejection and enabling personalized therapies with autologous cells. Their pluripotency allows differentiation into multiple cell types essential for vascular repair, and they serve as a valuable tool for studying AAA mechanisms and testing therapeutic interventions^51^. The ease of use and potential benefits of iPSCs does depend on the stage and type of differentiation, delivery method, and other therapeutic design components.

The challenges with iPSC-based therapies include their tumorigenicity (i.e. teratoma formation), for which some mitigation strategies include ensuring full removal of undifferentiated iPSCs and stem cell-like intermediates; the genetic heterogeneity and potential for genetic instability and mutations; and the technical complexity of reprogramming and differentiation protocols^53^. One particular major hurdle to efficacious use in therapeutics is incomplete maturation of iPSC-derived cells, for which many efforts are underway to improve^53^. iPSC-derived cells also have shown poor transplant engraftment and limited therapeutic response, making cell delivery and integration in the body another challenge^53^. Additionally, as with other cell therapies, scalability and quality control remain important challenges to consider.

## Preclinical trials of stem cell therapies

### MSC localized delivery

Compared to systemic delivery, MSCs have also been locally delivered in preclinical models of AAA (Fig. 1B). As an example of local delivery to the site of the aneurysm, BM-MSCs were generated into cell sheets and wrapped around the adventitial region of Ang II-infused AAs of ApoE^−/−^ mice^39^. The authors found that infrarenal diameter of the BM-MSC group was significantly smaller than the control aneurysm group at 4 weeks, and not significantly different from the sham group of ApoE^-/-^ mice that did not have induced aneurysm, while phrenic and ascending diameters had similar diameters of both aneurysmal groups, significantly increased compared to the sham group^39^. There was also significantly more elastin deposition in the aneurysms of mice that were treated with BM-MSC sheets, than in the AAA control group, and the degree of elastin deposition was not different when compared to the sham group^39^. MMPs, including MMP-2 and 9, IL-6, MCP-1, and TNF-α were significantly decreased in the MSC group, while the expression of IGF-1 and TIMP-1 were up-regulated, further evidencing the positive effects of BM-MSCs on AAA at the molecular level similar to other studies^39^. In another example, BM-MSC-seeded guinea pig xenografts were implanted in a rat model, in which BM-MSCs (10^6^) decreased AAA diameter growth more effectively than (5 million) VSMCs or AAA controls (infused only with culture media)^43^. Additionally, BM-MSCs decreased MMP-9 expression and macrophage infiltration and increased TIMP-1, compared to controls^43^. Interestingly, while neither BM-MSCs or VSMCs initiated repair of ECM in the medial wall layer, the BM-MSCs seemed to induce the accumulation of α-smooth muscle actin expressing cells surrounded by a collagen and elastin ECM^43^. This differs from the effects of more mature MSCs^43^, and shows the regenerative potential in effectively healing AAA.

Towards scaling to larger animal models, Turnbull et al. tested the therapeutic benefit of autologous implantation of BM-MSCs in a porcine model of AAA that was induced with an angioplasty balloon and collagenase type 1^42^. Following injection of 10^7^ BM-MSCs directly into the vessel wall, it was observed that the levels of VEGF increased at 72 hours after cell implantation, but returned to control levels at one week^42^. There was also increased capillary density in AAA tissues, but therapeutic benefits on aortic expansion was not described^42^. The results of these studies showed that induced aortic dilation was attenuated by BM-MSCs potentially through inflammatory and MMP modulation^42^. These results show that BM-MSC therapy abrogates dilation of the aorta, preserves elastin content and reduces MMP-2 and MMP-9 activity. These results indicate the potential of localized BM-MSCs as a treatment for halting aneurysm progression as evidenced in small-animal models but yet to be shown in larger models. These findings are supported by Li et al.^54^., who reported that MSC intervention across 18 studies is associated with reduced aortic diameter enlargement, reduced elastin degradation, and inflammatory cytokines. However, a drawback of localized cell treatment is the involvement of direct injection or placement of cells to the vessel itself, which may require invasive surgical implantation and thus may not be optimal for clinical translation.

### MSC systemic delivery

There have been studies of MSCs through intravenous administration, as it this can be less invasive, compared to localized delivery, while allowing the possibility for MSC homing to the site of the aneurysm^41^. Intravenous injections containing one million to three million cells each have been reported, ranging from single to multiple injections over the course of the study. The result of these experiments showed the attenuation of aneurysm dilation and formation even after one injection of one million cells after 14 days^41^. Sharma et al. found that treatment with human PL-MSCs (10^6^ injected on day 1) on mice showed suppression of mononuclear cell proliferation and proinflammatory cytokine IL-17 production (which promotes vascular inflammation and atherosclerosis, with proven effects in AAA also in this study), attenuating AAA formation^34^. There is evidence that MMP-2 and MMP-9 is decreased due to MSC treatment^41^. In a related work, Yamawaki-Ogata et al.^55^ reported that the pro- and active forms of MMP-2 and MMP-9 were significantly reduced after two weeks of delivery, but not significantly reduced at later time points, suggesting limited early-term benefits.

To examine the basic signaling mechanism of UC-MSC therapy, another study evaluated the role of NADPH oxidase, which is upregulated in human AAA, through HMGB1 (high mobility group box 1)^56^, suggesting a pro-inflammatory role^57^. HMGB1 is a damage associated molecular pattern molecule secreted rapidly by activated macrophages that stimulates IL-17^56^. HMGB1-blockade has been shown to suppress the development of AAA formation in animal models, reducing infiltration of macrophages and reducing MMP activity^57^. UC-MSCs showed inhibition of NADPH oxidase activation in macrophages, which attenuates HMGB1 production^56^. The authors showed a significant increase in HMGB1 production with the formation of AAA. NADPH oxidase 2 (Nox2) knockout mice with induced AAA had reduced HMGB1 expression, MMP-2 and MMP-9 activity, pro-inflammatory cytokine activity, and a decreased aortic diameter compared to wildtype mice with induced AAA^56^. This work demonstrates the intricate signaling pathways that mediate the effect of MSCs on AAA progression.

Similar to the localized treatments, IV delivered MSCs appeared to attenuate aneurysm expansion, and protect against elastin degradation. Wen et al. investigated intravenously injected (10^6^) umbilical-cord MSCs (UC-MSCs) into a AAA rat model^58^. UC-MSCs showed attenuation of aneurysmal expansion, reduction of elastin degradation and fragmentation, inhibition of MMPs (pro and active MMP2 and MMP9) and TNF-α expression^58^. The VSMC healthy contractile phenotype was preserved and/or restored in AAA as well, showing positive effects on the existing VSMCs and phenotype plasticity in the environment, further evidencing benefits of MSC therapies^58^.

Together, these studies, summarized in Table 2, demonstrate that systemic delivery of MSCs can attenuate aneurysm expansion, reduce inflammation, inhibit MMP activity, and promote elastin preservation, contributing to overall AAA stabilization. Compared to localized delivery, MSCs can be delivered systemically using less invasive means to deliver cells for treatment. However, some of the limitations of systemic delivery include dilution of cells, non-specific distribution, and potential sequestration of MSCs in off-target tissues, which may reduce their effectiveness at the aneurysm site. Additionally, there is evidence that a substantial number of cells are trapped in other tissues before reaching the aneurysm, potentially reducing the effects^55,59^.

**Table 2:** MSC therapies for AAA models

Disease model	Cell type	Delivery Method	Day(s) of MSC Delivery	Day(s) of Harvest	Output Measurement	Reference
Angiotensin II in ApoE mice	Murine BM-MSC	Multiple IV injections	Days 0, 7, 14, 21	Day 28	Decreased aortic diameter in infrarenal regions; elastin content of MSC group significantly higher than control, also suppressed inflammatory response and MMP activity	^41^
Elastase model in mice	Human umbilical cord-MSC	IV injection	Day 0	Day 14	MSC anti-HMGB1 treatment attenuated AAA formation IL-17 production and inflammatory infiltration	^56^
Collagenase and elastase model in pigs	Porcine BM-MSC	Direct injection into aortic wall	Day 1	Days 1, 3, 7	Expressed high levels of VEGF in MSC group	^42^
Elastase model in mice	Murine BM-MSC	IV injection	Days 1, 3, 5	Day 14	Both Male and Female MSC decreased aortic dilation TNF-α, IL-1β, and MCP-1 were only decreased with female MSC	^105^
Angiotensin II in ApoE mice	Murine BM-MSC	Cell sheets	Day 0	Day 28	MSCs decreased active MMP-2, preserved elastin content and AA development was site specifically inhibited	^39^
Angiotensin II in ApoE mice	Murine BM-MSC	IV injection	Day 28	Days 42, 56, 84	No significant difference at 8 weeks for aortic diameter. Pro and active mmp2/9 were significantly reduced in MSC groups at week 2, elastin in the media was preserved at all three timepoints. No statistical difference between IL-1β, TNF-α, TGF-β1	^55^
Guinea pig xenograft in rat	Rat BM-MSC	Direct injection to aneurysm	Day 14	Days 21, 42	MSCs decreased AAA expansion compared to culture medium infusion, decreased expression of MMP-9 and macrophage infiltration	^43^
Elastase-induced rat model	Human UC-MSCs	IV injection	Day 0	Day 14	The treated group demonstrated effectively decreased AAA expansion, with increased elastin preservation and contractile VSMC marker expression and decreased pro and active MMP2 and MMP9 expression and TNFα	^58^
Calcium chloride and elastase model in mice	Multilineage-differentiating stress-enduring cells isolated from human BM-MSCs	Multiple IV injections	Days 0 (3 days post aneurysm induction), 7, 14	Days 21, 56	At 8 weeks no difference between single injection vs multiple injections of muse cells; both significantly smaller diameters than the non-muse vehicle and MSC groups; multiple injection group had a higher ratio of elastin content than the non-muse vehicle and MSC groups; all groups had significantly lower F4/80+ cells than the vehicle	^59^

*AAA* Abdominal Aortic Aneurysm, *IV* Intravenous, *BM* Bone Marrow, *MSC* Mesenchymal Stem Cell, *WT* Wild Type, *ApoE* Apolipoprotein E, *IL* Interleukin, *TNF* Tumor Necrosis Factor, *TGF* Transforming Growth Factor, *MMP* Matrix Metalloproteinases

### ADSC therapy

As an alternative to BM-MSCs, studies have been conducted using ADSCs to look at their effect on AAA. Blose et al. delivered ADSCs (10^5^ cells) using a port targeting the periadventitial aneurysm in a mouse model^60^. After two weeks, the aortic diameter and elastin content of the treated group was similar to that at 5 days, when the treatment was administered, suggesting that localized ADSC therapy abrogated the progression of the disease^60^. In another study, 4×10^6^ ADSCs were injected systemically through the common carotid artery in a rat model^61^. Assessment of the elastin fibers showed no significant difference between ADSC or BM-MSC groups^61^. However, protein expression of elastin was significantly higher in the ADSCs than the BM-MSCs and control at 21 days^61^. Gene expression of elastin demonstrated a significant increase over time with the ADSCs group^61^. Active and pro forms of MMP-2 and 9 in the ADSCs group were lower than in the sham or control groups^61^. Gene expression of MMP-2 and MMP-9 in SMCs increased then plateaued after 48 hours with a subsequent decrease, illustrating ADSCs’ inhibition of MMP secretion^61^. This suggests a beneficial role in the reconstruction of elastic fiber and modulating the MMP pathway, both of which are shown to slow or prevent AAA growth.

However, the beneficial effects of ADSCs appear to be mixed and may be dependent on the frequency or timing of cell delivery. When comparing the efficacy of ADSCs to BM-MSCs on AAA physiology, the authors reported that ADSCs supported higher in vitro protein expression for elastin, but in vivo there was no significant difference between the two cell therapies^61^. In another study^62^, 10^6^ “adipose-derived mesenchymal regenerative cells” from male Sprague Dawley rats were intravenously injected in a rat model. The authors demonstrated that these autologous rat ADSCs were not able to attenuate AAA progression, with no significant difference between the saline control and ADSC group in neutrophil or macrophage infiltration, elastin content, or aortic diameter at day 28^62^. The authors speculated that additional dosages could be needed to have an effect^62^. In contrast, another study having weekly MSC IV administration reported positive results and effective attenuation of AAA in a mouse model^41^. These reports of mixed results highlight the need to optimize and then standardize cell dosing for each cell type. Together, these studies (Table 3) illustrate the therapeutic benefit of ADSCs for treatment of AAA.

**Table 3:** ADSC therapies for AAA models

Disease model	Cell type	Delivery method	Day(s) of ADSC delivery	Day(s) of harvest	Output measurement	Reference
Elastase model in mice	Mouse ADSCs	Subcutaneous microport delivery	Day 5	Days 5, 14	Cell treated aneurysms had less dilation and intact elastin	^60^
Elastase model in mice	Human ADSCs	IV injection	Day 0	Days 1, 4, 7, 14	ADSCs suppressed AA expansion through 14 days, increased Treg, decreased levels of CD115 + CXCR1 − LY6C+ monocytes,	^49^
Calcium chloride model in rats	Rat ADSCs	IA injection	Day 0	Day 28	In vivo ADSC group significantly increased elastin production, decreased MMP2/9	^61^
Dacron patches in pigs	Pig ADSCs	Catheter scaffold with fibrin sealant	Day 0	Days 1, 7, 21, 35, 49, 63	MSC group has lower inflammation reaction, and observed GFP marked cells up to 3 weeks	^44^
Calcium chloride and elastase model in rats	Rat ADSCs	Cell-seeded scaffold	Day 0	Day 14	ADSC group diameter didn’t differ significantly from the sham group and had better looking elastin than the aneurysm controls. The cell group had a significantly higher number of VSMCs in the vessel and lower levels of macrophages	^81^
Elastase model in rats	Rat ADSCs	IV injection	Day 0	Day 28	No significant difference between cell and control group in neutrophil or macrophage infiltration, elastin content, or aortic diameter.	^62^
Elastase + collagenase injected in pigs	Human adipose-derived MSCs	GelFoam sponge directly applied	Day 0	Day 21	Reduced aortic dilation, higher preservation of elastin, preserved SMCs, increased VEGF, TIMP1, and TIMP3, and Young’s elastic modulus. Higher collagen content, alpha smooth muscle action, and elastin perturbation than untreated.	^82,83^

*AAA* Abdominal Aortic Aneurysm, *ADSC* Adipose-derived Stem Cells, *IV* Intravenous, *IA* Intraarterial; *MSC* Mesenchymal Stem Cell, *WT* Wild Type, *ApoE* Apolipoprotein E, *IL* Interleukin, *MMP* Matrix Metalloproteinases, *Treg* Regulatory T Cells, *GFP* Green Fluorescent Protein, *VSMC* Vascular Smooth Muscle Cells, *PPE* Porcine pancreatic elastase, *SMC* Smooth Muscle Cells, *VEGF* Vascular Endothelial Growth Factor, *TIMP* Tissue Inhibitor of Matrix Metalloproteinase.

### iPSC-based therapy

Although iPSCs have been investigated for different use cases, such as examining their differentiation into aortic SMCs for modelling Marfan syndrome in mice^63^, their preclinical investigation as a therapeutic is limited. An early study from Mulorz et al., described above, that compares iPSC-derived vascular smooth muscle progenitors with VSMCs in mice showed that these iPSC-derived cells can be delivered using scaffold-based approaches, localize to the aneurysm wall, and support native SMC retention^28^. However, the primary VSMCs were more effective therapeutically, causing more significantly decreased AAA growth^28^. Given the many potential advantages of iPSCs however, both biologically and in terms of clinical translation and scalability, further preclinical investigation is necessary to fully explore this potentially promising cell source.

## Clinical trials of stem cell therapies

Planned clinical trials for cell therapies for treatment of AAA have been initiated to explore the utility of MSC treatments for AAA (Fig. 1C). The STOP-AAA trial studied autologous BM-MSCs in attenuating the expansion of small aneurysms^64^. Three doses of 2 × 10^6^ MSC/Kg or placebo were delivered at baseline, 24, and 53 weeks with the experiment planned to end at 18 months^64^. No update on this trial has been reported. As of December 2024, there were two relevant cell-therapy related clinical trials with activity since 2020 on ClinicalTrials.gov to the knowledge of the authors. First is the “VIVAAA/ARREST” trial (ClinicalTrials.gov ID NCT02846883) from Indianapolis, U.S.A., which sought to inject allogeneic MSCs to stimulate T-regulatory cells via IV injection in patients with small AAA and evaluate therapeutic safety and efficacy^65^. This was with the goal of determining if MSCs can decrease inflammation and slow aneurysm growth. Interventions included intravenous infusion of 1 million MSCs/kg, 3 million MSCs/kg, and a placebo^65^. First posted in 2016, this study was terminated in September 2021 due to slow enrollment after being put on hold during the COVID-19 pandemic^65^. The second trial from Madrid, Spain first posted in July 2024 (ClinicalTrials.gov ID NCT06488898) evaluates AAA stabilization using allogeneic adipose-derived MSCs (ADSCs) injected locally in the aneurysmal sac, with methods of MSC administration described previously^44,66^. Currently recruiting and with a target completion date for data collection of December 2025, if completed successfully, this trial should demonstrate the outcomes in humans of cell therapy products in AAA, particularly MSCs, providing the first major clinical outcomes in this area^44,66^. Results from these ongoing trials will reveal the plausibility of these therapies in humans as well as investigate and translate the findings from the many preclinical studies.

## Cell-derived products for AAA treatment

**Fig. 3:**
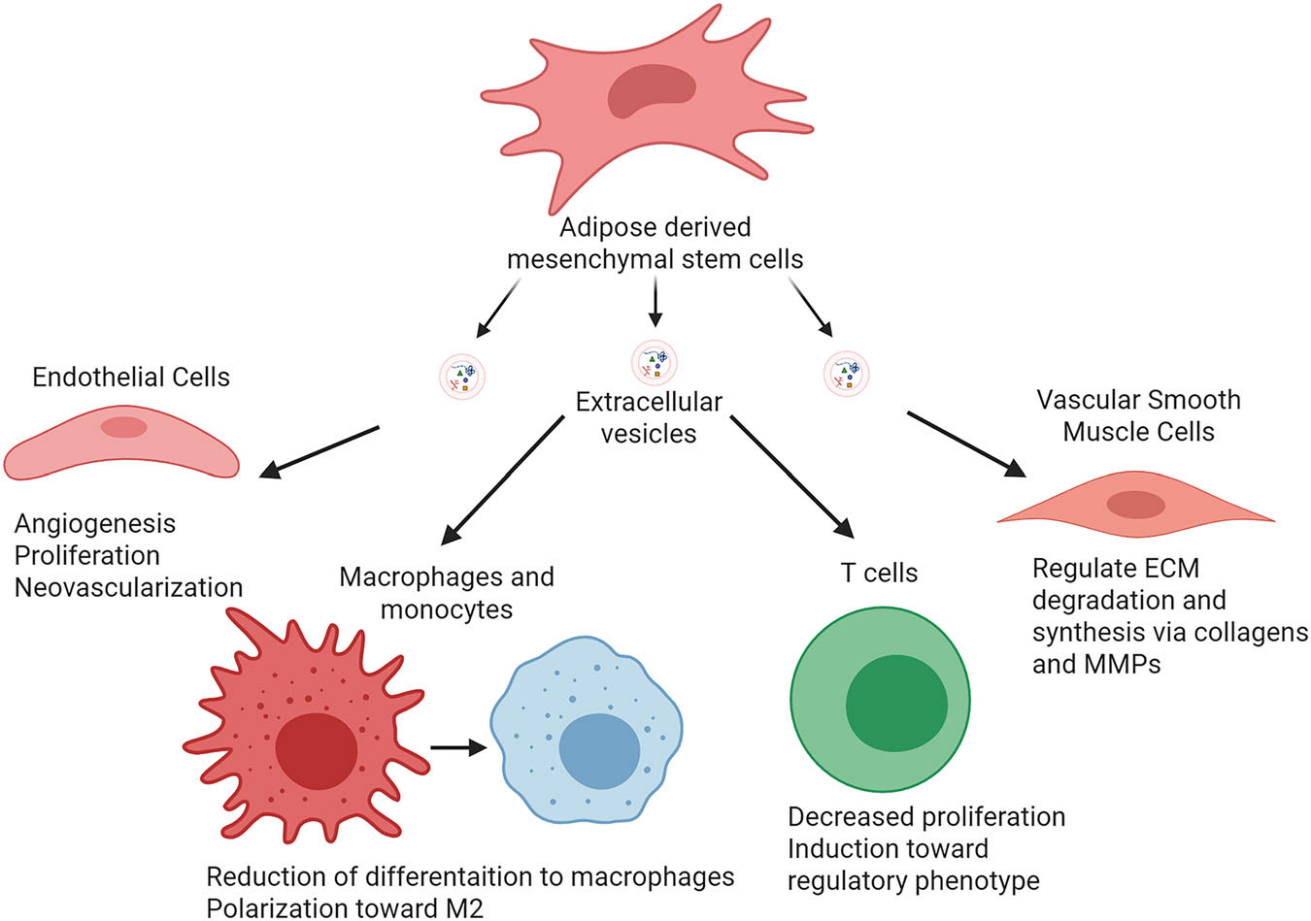
Overview of cells that can be affected by MSC-EVs in relation to AAA. VSMCs, immune cells (T cells, macrophages, and monocytes), and endothelial cells are depicted. Created in BioRender. Marini, A. (2025) https://BioRender.com/h8r5hjb.

As cell therapies can carry regulatory hurdles, recent studies have pursued cell free methods as an alternative. Included within these cell free methods are EVs (Fig. 3)^67–70^. Spinosa et al. utilized human umbilical cord from Wharton’s Jelly MSC derived EVs in a mouse model of AAA^71^. EVs are nano-sized, membranous structures containing cellular components and surface markers (e.g. proteins, mRNAs, miRNAs, DNA, and lipids) that are secreted into the extracellular space under healthy and pathological condition^72^. The importance of EVs in cellular communication and other processes makes them relevant and promising as biomarkers and in therapeutics^72^. In this study, both MSCs and EVs had decreased AAA expansion compared to untreated controls^71^. They also had decreased inflammatory cytokines and better preservation of elastin compared to untreated controls^71^. Their study also investigated miR-147 as a prominent microRNA for reducing inflammation. In another study, Chen et al. intravenously injected umbilical cord MSC-EVs into a mouse model of AAA and saw decreased aortic diameter expansion and increased elastin preservation compared to controls^73^. Kozakai et al. used mouse derived bone marrow MSC-EVs for intravenous injection into a mouse model of AAA^74^. The bone marrow MSC-EV treated group had decreased diameter expansion, decreased pro and active MMP2 and MMP9 activity, increased elastin preservation, decreased F4/80+ macrophages, and increased CD206+ macrophages compared to the controls^74^. Additionally, analysis of the MSC-EVs revealed they contained inhibitory microRNAs of aneurysm formation^74^, implicating that these EVs would cause the downstream effects observed. Hu et al. used mouse adipose MSC-EVs as a delivery system of miR-17-5p via an intravenous injection into a AAA mouse model^75^. With these EVs, there was increased elastin preservation, decreased inflammatory cytokines, decreased aneurysm expansion all of which was further enhanced by miR-17-5p^75^. They also tracked their EVs and saw lots of accumulation in the liver with some accumulation in heart, spleen, kidney, and lung^75^.

There have also been some in vitro studies performed with EVs^76–78^. In one study by Cunnane et al., adipose MSC-EVs stimulated elastin and collagen production in 3D SMC seeded fibrin gel constructs^76^. Sajeesh et al. performed two different EV studies. In the first study, human bone marrow MSC-EVs were used for treatment of elastase damaged SMCs^77^. The EVs decreased MMP2 and increased TIMP1 and 2 gene expression, decreased active MMP2 and overall MMP protein level, increased TIMP2 level, decreased MMP2/TIMP2 ratio, decreased MMP2 activity, increased elastin (ELN), fibulin 5 (FBLN5), lysyl oxidase (LOX) gene expression, increased LOX protein, and increased elastin content^77^. In another study, human BM-MSC-EVs were functionalized with cathepsin targeting molecule, as an eventual means for targeting AAA^78^. These EVs decreased MMP2 expression and increased LOX expression in elastase damaged SMCs^78^. The functionalization also improved EV uptake^78^. These studies indicate the regenerative potential of MSC EVs for treatment of AAA. Circulating miRNAs have also been investigated as markers particularly for early-stage diagnosis^79^. While more is being investigated, 9 key miRNAs in related pathways (e.g., vascular inflammation, VSMC phenotype switching, ECM degradation, etc.) are implicated in known AAA mechanisms^79^.

## Biomaterials-based approaches for AAA treatment

The development of a stem-cell based therapeutic targeted at both the early and late-stage AAA treatment requires a combination of novel and continuously emerging bioengineering strategies and techniques. The integration of biomaterials and scaffolds to enhance stem cell delivery for to create aortic grafts is a major area of interest. Depending on the biomaterial and its associated or designed properties (e.g., ceramics, natural polymers, and synthetic polymers), different characteristics can be exploited for more optimal delivery and cell survivability^80^.

ECM patches have been tested for the delivery of therapeutic cells to the peri-adventitial space of the aneurysm. For example, Parvizi et al. performed an intraluminal infusion of elastase into rats. A recombinant collagen peptide patch was seeded with 2×10^6^ ADSCs^81^. After two weeks the ADSC group diameter didn’t differ significantly from the sham group and also had better looking elastin than the aneurysm controls^81^. The cell group had a significantly higher number of SMCs in the vessel as well as lower levels of macrophages^81^. In larger animals, Zilberman et al. delivered 10^6^ human ADSCs suspended in GelFoam (an absorbable gelatin sponge^82^) placed peri-adventitially on a pig model AAA, induced via elastase and collagenase I^82^. In the stem cell-treated group, there was reduced aortic dilation, higher preservation of elastin, preserved SMCs, and increased VEGF (vascular endothelial growth factor), TIMP1, and TIMP3 in the aorta^82^. The untreated group saw significantly decreased collagen content, α-smooth muscle actin, and elastin perturbation^82^. Kooragayala et al. expanded upon this work with the same porcine model, cell type, and GelFoam delivery and saw increased Young’s elastic modulus (ability to withstand stress) as well as preservation of elastin in the treated group, showing efficacy and promise of ADSCs in larger pig models^83^.

Besides the use of biomaterials for therapeutic cell therapy, there is also a rise of biomaterials to be used for generating vascular grafts. To address the concerns of EVAR procedures leading to due to endoleaks and limited long-term device durability, several bioengineered stent grafts have been investigated focusing on improved tissue integration post-EVAR^84^. Takeuchi et al. reported that polyethylene terephthalate/polyglycolic acid-based grafts in mongrel dogs promoted both histologic and mechanical integration by recruiting the host tissue into the graft scaffold, thereby preventing potential postoperative migration and endoleaks^85^. Furthermore, through two consecutive studies, Kawajiri et al. demonstrated the application of in-body tissue architecture technology to fabricate self-expandable aortic stent allografts^86,87^. An acryl/nitinol mold was subcutaneously embedded into beagles for four weeks to produce an autologous implantable collagenous tissue with the desired shape^86^. Subsequently, the structure was harvested and implanted as allograft into the infrarenal abdominal aorta of the animal. The implanted stent grafts were observed to be integrated with the native aorta within a month while exhibiting excellent neo-endothelialization^87^. These studies highlight the emerging area of biomaterials for co-delivery of therapeutic cells or for the generation of vascular grafts to treat AAA.

## Clinical translation barriers and considerations

Clinical translation of cell-based products is challenging, from the complex biological environment in the human body, to real-world barriers in manufacturing and scalability, and the importance of quality control and regulatory compliance. Translation not only requires resolving technical challenges and current limitations, as described below, but also building and fitting into robust translational frameworks (e.g. regulatory, manufacturing) in a cost-effective way.

Biologically, clinical integration is complicated by factors like variability in cell quality, survival, engraftment, and functional maturation. For stem cell and cell-based technologies, risks such as tumorigenicity, off-target differentiation, and unexpected immune responses remain major safety considerations^88^. Stem cells also face specific challenges with differentiation needing to yield mature and stable cell populations at scale^89^. Long-term functional integration and effects in humans are critical to elucidate as technologies move to clinical trials and beyond. Moreover, working closely alongside and in adherence to regulatory bodies is critical to safely and effectively bringing these technologies to patients with regulatory approval. Consideration of validated clinical trial endpoints and evaluation criteria for therapeutics, like in earlier-stage AAA, is also important to ensure outcomes reflect the true biological effects being investigated.

Stem cell-based products face unique quality control and manufacturing considerations, including the need for reproducible large-scale production under good manufacturing practice (GMP) standards and authorization, validated potency assays, and strict release criteria to ensure batch-to-batch consistency^90,91^. Moving from smaller-scale academic studies to clinical-grade manufacturing requires addressing hurdles in cryopreservation, storage stability, and distribution, while maintaining phenotype and function. As this and other supporting technology has improved, the translation of stem cell technologies has become increasingly possible and scalable.

There are significant costs and strategic trade-offs throughout the translation of stem-cell technologies that shape product design choices and implementation. Decisions such as autologous vs. allogeneic sources, selection of cell type and harvesting, and complexity of the delivery mechanism must be balanced with scalability, manufacturing, cost-effectiveness, and the realities of adoption into healthcare systems, all critical determinants of successful clinical translation. This necessitates comprehensive and early consideration of the trade-offs and eventual clinical implementation to enable these therapies to successfully reach patient populations in the face of so many barriers. Clinical success becomes more likely with rigorous quality controls, effective scalability and manufacturing, demonstrated durability of benefit, real-world economic viability, and alignment with regulatory pathways, in addition to strong scientific development and testing. Iterative feedback between preclinical studies and early clinical trials will be essential to first identifying and improving upon approaches with true therapeutic and clinical promise for AAAs.

## Current limitations and future perspectives

As illustrated throughout this review, the AAA field is witnessing significant innovation across diagnostics, therapeutics, monitoring, and understanding its pathophysiology. Key areas requiring further exploration include the identification of optimal cell sources. While MSCs, ADSCs, and VSMCs have been widely studied, iPSC-derived SMCs present a novel, patient-specific option with reduced immunogenicity and enhanced differentiation potential. Comparative studies are needed to assess their relative efficacy while using consistent models. Furthermore, additional factors such as ease of harvesting, cell stability, and regulatory approvals must also be considered while evaluating their translational ability.

Another critical issue is the limited control over the differentiation pathways and long-term fate of administered stem cells, particularly with respect to off-target differentiation that could compromise therapeutic efficacy or induce adverse effects. For example, MSCs have demonstrated a tendency toward osteogenic differentiation, which is undesirable in the vascular environment and could exacerbate vascular calcification. Similarly, iPSC-derived cells, while offering patient-specific advantages, carry risks of incomplete or aberrant differentiation, potentially leading to tumorigenicity or fibrosis. Moreover, most existing studies on AAA treatment have reported limited data on the biodistribution and long-term response of the transplanted cells, leaving significant gaps in understanding their mechanisms of action and safety profile. Therefore, future studies should address these shortcomings through rigorous assessments of cell engraftment, fate, and genetic stability using standardized in vivo tracking methods, along with stringent quality control, to enable targeted repair and regeneration of the aortic tissue.

Another key consideration is the optimization of dosing, timing, and delivery methods. The therapeutic efficacy of cell-based treatments is influenced not only by the number of cells administered, but also by when and how they are delivered. Preclinical studies highlight the critical importance of timing, with administration after aneurysm formation better reflecting the clinical context and yielding more translatable outcomes compared to interventions initiated at model induction. Furthermore, while systemic administration is less invasive and potentially more scalable, it is limited by poor cell localization and significant off-target effects. In contrast, targeted delivery methods, such as direct injection into the aortic wall using hydrogel-based systems, offer improved therapeutic performance by ensuring higher cell retention and localized effects. Future work should integrate optimal dosing with post-aneurysm timing and localized delivery strategies to validate the clinical potential of stem cell-driven AAA treatment.

Additionally, the lack of studies that investigate sex-based differences in AAA models and stem cell therapies limits critical insight into how the treatments would behave in male and female patients. The majority of studies discussed in this review only used male animals or cells from male animals or human male patients. While AAA mostly affects men, women have worse outcomes related to rupture and surgical repair^92^. These worse outcomes underscore the need to investigate the effects of stem cell treatment on both male and female animals, as there has been evidence that the sex of the animal affects AAA progression in vivo^93–95^. Also, there could be sex-based differences in the cell source for treatment^96,97^. Thus, it is necessary to further investigate the effects of sex of the animals and sex of the cell source on the treatment of AAA.

In the future, omics and high throughput techniques (e.g. proteomics, single-cell RNA sequencing) will provide deeper insights into cell therapy-induced changes. These approaches have enabled the discovery of relevant biomarkers and will help validate the therapeutic efficacy of these approaches. Single-cell RNA sequencing provides granular insights into how different cell types in the aortic wall will respond to the applied treatments^98^. Furthermore, integration with spatial transcriptomics enables an understanding of gene expression within tissue sections, thereby revealing the underlying cellular interactions^99^. Proteomic analyses can identify the interactions, function, composition, and structure of proteins and their activities, responsible for ECM remodeling and inflammation reduction, as described by Al-Amrani et al.^100^. Combining these methods with advanced computational tools and machine learning, provides a more intricate understanding of cellular and tissue mechanisms in AAA for development of advanced therapeutic approaches.

With the rapid advancement of artificial intelligence (AI), it is increasingly applied to improve AAA outcomes as well. Besides ‘omics, AI has proven especially valuable in boosting the current imaging and monitoring technologies. Ongoing clinical trials are evaluating its usage for better aneurysm modelling for predictive rupture and enhanced monitoring. These include: the VASCUL-AID-RETRO trial, using AI for vascular disease risk and progression prediction (NCT06206369)^101^; the ART in EVAR trial, predicting AAA shrinkage following stent-placement with AI (NCT06250998)^102^; the ViTAA registry pre- and post-operative monitoring for EVAR using aortic mapping technology (NCT05004051)^103^; and the IAVASC trial, implementing AI into automatic analysis of vascular network segmentation to predict risk for AAA (NCT06451315)^104^, with the ViTAA and IAVASC trials using AI technology developed previously^103,104^. These efforts aim to enhance predictive modeling, monitoring, and decision-making, enabling more proactive care for surgical treatment.

In the immediate term, more efforts should be focused on working towards the clinical stage by validating current findings and hypotheses in human studies. Additionally, future research should explore the potential for combinatorial approaches. Integrating cell therapies with biomaterials has shown evident benefits in the delivery of the regenerative effects. How these cells may interact and be enhanced by pharmacological agents for example would be beneficial to investigate for how they could enhance therapeutic efficacy and durability. For example, combining MSCs with matrix-modifying enzymes or anti-inflammatory drugs may synergistically enhance ECM regeneration and reduce aortic wall stress. Additionally, cell-derived products, such as exosomes, will be interesting to further investigate and ascertain as potential therapeutic candidates.

Overall, stem-cell-based therapies hold significant promise for the treatment of AAA, with numerous studies evidencing the therapeutic effects and benefits of cell-based therapies. However, their full clinical potential has yet to be realized. As research in this area expands, clinical trials are necessary to understand the effects of these cells in humans and eagerly awaited. Addressing current limitations through rigorous investigation of cell sources, dosing, timing, and delivery methods, alongside leveraging advanced technologies like omics, will be crucial for translating these therapies into more effective treatments for AAA.

## Data Availability

All data described here are cited. No new data was generated.
